# “I Gave Up Football and I Had No Intention of Ever Going Back”: Retrospective Experiences of Victims of Bullying in Youth Sport

**DOI:** 10.3389/fpsyg.2022.819981

**Published:** 2022-02-15

**Authors:** Xènia Ríos, Carles Ventura, Pau Mateu

**Affiliations:** ^1^Institut Nacional d’Educació Física de Catalunya (INEFC), Universitat de Barcelona, Barcelona, Spain; ^2^Grup d’Investigació Social i Educativa de l’Activitat Física i de l’Esport, (GISEAFE), Barcelona, Spain; ^3^Universitat de Barcelona (UB), Barcelona, Spain

**Keywords:** bullying, youth sport, retrospective experiences, victims, sport club

## Abstract

Bullying is a global issue that, beyond school, is present in different social contexts, such as sport environments. The main objective of this study was to get to know the experiences of victims of bullying in sport throughout their youth sport training. Semi-structured interviews to four Spanish women and seven Spanish men were carried out, within an age range of 17–27 (*M*_*age*_ = 21 years, *SD* = 3.69). The following main themes were established by means of a hierarchical content analysis: (a) “bullying characterization,” (b) “dealing with bullying,” and (c) “consequences of bullying.” The results show the presence of physical, verbal and social bullying in the sport context, with the changing room being the space where this type of behavior is most frequently developed. Most victims show an internal attribution (self-blame) for the bullying event, related to their motor skills and their personal physical and psychological characteristics. Double victimization can be observed, at the sport club and at the educational center. Passive strategies are used to deal with the situation, while little support is shown by sport agents (teammates and coaches). The victims, as a consequence of the bullying experience, suffer from short and long-term negative effects on a psychosocial level. The study highlights the necessity to design and implement programs focused on the prevention, detection and intervention of bullying for sport organizations, bearing in mind all the agents that make them up (coaches, management teams, families, and players). Furthermore, the importance of promoting the creation of safe sport environments, free from any kind of violence, is emphasized.

## Introduction

The International Society for Physical Activity and Health (ISPAH) mentions sport practice as one of the best “investments” to promote the practice of physical activity among citizens (Milton et al., 2021). Although differences are observed between countries around the world (Tremblay et al., 2016), in Europe approximately two out of three children and adolescents perform some type of sport practice at sport clubs (Kokko et al., 2019). Studies show that the sport context becomes an adequate space to promote a correct physical, psychological and social development among its participants (Banjac et al., 2020). In addition, sport practice during childhood will predict optimal physical activity habits during adulthood and an improvement in their cardio-metabolic state (Murphy et al., 2016; Logan et al., 2020). At the same time, it is customary to relate it to the development of a series of “positive” behaviors and values, although sport practice it is not, *per se*, good or bad (Banjac et al., 2020). Thus, while pro-social behaviors may occur, it might enable the emergence of an environment in which negative behaviors can be normalized and the appearance of situations such as bullying can be favored (Logan et al., 2020; Milovanović et al., 2020).

Bullying is defined as a set of negative behaviors, with the intention of harming a victim, carried out repeatedly over time and involving an imbalance of power or strength between those involved (aggressor-victim) (Olweus, 1994). It can be classified according to traditional bullying and cyberbullying (Chu et al., 2019), with a prevalence of 35 and 15%, respectively (Modecki et al., 2014). Traditional bullying includes the typologies of physical (hitting, pushing, damaging belongings…), verbal (insulting, name-calling, making fun of others…) and social bullying (excluding, ignoring…) (Menesini and Salmivalli, 2017). At the same time, these typologies can present a direct interaction (face to face between the aggressor and the victim) or without explicit interaction between them (Olweus, 2006). Cyberbullying is a type of bullying through the use of mobile phones or the Internet (Smith, 2019b) and is characterized by the possibility the aggressor has to hide their identity, as well as by the rapid dissemination of the contents offered by the virtual space (Smith et al., 2008). Bullying is understood as a social process, and cannot be reduced to a bidirectional dynamic between the aggressor and the victim (Salmivalli et al., 1996), since the behavior adopted by “equals” will be of great importance to maintain or inhibit these situations (Nocentini et al., 2020). Regarding the health effects of being a victim of bullying, the appearance of problems related to anxiety, depression, non-suicidal self-harm, suicidal ideation, suicide attempts, substance abuse, decreased academic performance, social isolation and psychosomatic alterations is observed (Moore et al., 2017). These effects are important in the short term, especially during childhood and adolescence, and persist throughout adulthood (Arseneault, 2018; deLara, 2019; Camodeca and Nava, 2020), although they tend to decrease in the long term (Schoeler et al., 2018).

Although research into the bullying phenomenon has been focused on the school since its early studies in the 1970s by Olweus (1978), bullying appears in other social contexts (Monks and Coyne, 2011), such as sport (Nery et al., 2019). The interest in research on bullying in the sport world is more recent and, above all, it has been devoted to the analysis of the prevalence of the victimization of the phenomenon, observing wide and dissimilar ranges that vary from 8.9% (Nery et al., 2019) to 48.8% (Mishna et al., 2019). This variability, as it occurs in the educational context according to Smith (2019a), may happen due to the use of different conceptual criteria on the phenomenon and dissimilar methodological elements used in each of the studies (Vveinhardt and Fominiene, 2019). In relation to the risk factors of becoming a victim of bullying in sport, the presentation of some type of disability (Danes-Staples et al., 2013), being overweight (Bacchini et al., 2015), belonging to ethnic minorities (Kentel and McHugh, 2015), the sexual orientation -homophobic bullying- (Baiocco et al., 2018; Denison et al., 2020) or having poor sport skills (Kerr et al., 2016; Mishna et al., 2019; Vveinhardt et al., 2019b; Flores et al., 2020) stand out. Boys present higher rates of aggression, but not victimization (Evans et al., 2016; Vveinhardt and Fominiene, 2019), the typology of verbal bullying is the most frequent and the changing room is the sport space where more bullying situations appear (Mishna et al., 2019; Nery et al., 2019; Flores et al., 2020). Regarding the coping strategies that victims adopt in bullying situations, Nery et al. (2019) observed, with a sample of male athletes that, above all, they used strategies focused on emotions, communicating the situation to the family and/or the coach. Stirling et al. (2011) highlight that traditional sport culture does not encourage victims to break the law of silence as it is considered a sign of weakness, which causes continuity in bullying behaviors by the aggressor (Vveinhardt et al., 2017). On the other hand, bullying in sport has a negative emotional impact on the victims (Kentel and McHugh, 2015), who tend not to enjoy the sport, to have low self-esteem (Jewett et al., 2019), to feel isolated from the rest of the team (Evans et al., 2016), to have a low athletic as well as academic performance (Vveinhardt et al., 2017; Jewett et al., 2019) and to change sport clubs or abandon sport practice (Evans et al., 2016; Nery et al., 2020).

In recent years, studies have appeared that have dealt with analyzing the phenomenon of sport bullying from focus groups with coaches, families, and athletes (Flores et al., 2020), through interviews with coaches (Vveinhardt et al., 2019b; Nery et al., 2020), athletes and former athletes (Nery et al., 2020), team sport captains (Kerr et al., 2016) and professional players (Newman et al., 2021). However, there has been little research looking at bullying situations among the victims themselves. Jewett et al. (2019) analyzed the negative emotional consequences of being a victim and Vveinhardt et al. (2019a) focuses on its nature, causes and consequences. Given the absence of research analyzing the phenomenon of bullying in sport in depth from the perspective of the victims themselves during childhood and/or adolescence, the purpose of this research was to determine retrospectively the experiences of bullying among athletes who had been victimized during their youth sport training. The obtained results may be of interest to the professionals in the sport environment (coaches, psychologists, sport leaders, etc.) as they will allow a more specific knowledge of the problem and the design of preventive, detection or intervention strategies adapted to the context.

## Materials and Methods

### Philosophical Underpinning

This descriptive and retrospective research is located in the post-positivist paradigm. According to Lincoln et al. (2011), post-positivism is adhered to the critical realistic ontology, suggesting the existence of an external and objective reality but, given that observations are fallible, it is assumed that this can only be apprehended from an imperfect way. Regarding the epistemological approach of post-positivism, the authors refer to a modified dualism/objectivism. Thus, it is understood that researchers may approach external reality, but the fallibility of their observations, as well as possible biases based on ideological positions and personal values, may affect the way in which they understand what is analyzed. Assuming the existence of an external and objective reality favors, according to Sparkes and Smith (2014), the formulation of stable guidelines that facilitate the control and prediction of phenomena.

### Participants

After disseminating the research through social media and emails, participants were recruited using a non-probabilistic sampling by criteria (Sparkes and Smith, 2014). At the same time, this sample was expanded with a snowball sampling (Parker et al., 2019). The inclusion criterion for the participants in this study was to have been victims of bullying in sport throughout their youth sport training. This criterion was assessed through a previous individual interview with the participants, where their experiences were assessed to be consistent with the characteristics of bullying established by Olweus (1994). A total of 13 people were interested in participating in the study. After reviewing the inclusion criterion, two potential participants were dismissed: one of them had suffered from bullying exclusively in the school environment, while the other one had been a victim of bullying by his coach and not by their equals. Finally, 11 people (four women and seven men) were considered valid to be part of the present study. At the time of the interview, participants were 17–27 years old (*M*_*age*_ = 21 years, *SD* = 3.69). When they experienced the bullying situation, they were between 10 and 16 years old and practiced individual and collective sports in various sport clubs in Catalonia (Spain). To ensure anonymity of the participants, they were assigned a pseudonym. Also, the data presented in the article are sufficient to answer the purpose of the research and, at the same time, do not allow the identification of the interviewees. Two of the participants had a disability: hearing disability (Martí) and visual disability (Diego). The characteristics of the participants are shown in Table 1.

**TABLE 1:** Participants of the study.

Pseudonym	Age at the time of the interview	Gender	Sport category at the time of victimization
Martí	22	Male	Football
David	18	Male	Basketball
Roger	22	Male	Football
Noa	17	Female	Basketball
Gerard	24	Male	Football
Carla	27	Female	Swimming
Ivan	18	Male	Basketball
Anna	27	Female	Basketball
Diego	18	Male	Swimming
Victor	18	Male	Basketball
Laura	20	Female	Athletics

### Instrument

For data collection, the authors chose to conduct semi-structured interviews (Sparkes and Smith, 2014). This method provided flexibility, as it allowed to change the order of the questions, making additional ones, and intentionally orienting the participants toward the goal of the research (Flick, 2009). At the same time it gave greater control to the interviewees, allowing them to explain their experiences in depth, in their own words and without limitations (Sparkes and Smith, 2014). Creswell (2012) declared that this data gathering method is particularly useful for exploring sensitive and emotional issues such as bullying victimization. An interview script was designed based on a review of the bullying literature in the sport context (Fisher and Dzikus, 2017; Nery et al., 2020). The interview consisted of an initial part where information about less sensitive personal issues was discussed, such as their first sport experiences. The main part of the interview followed, where the participants were asked to explain in detail their bullying experiences, such as the coping strategies used by the victims or the consequences of the experienced situation, among others.

### Procedure

The study participants were sent a written document of informed consent stating the purpose and procedure of the research and its voluntary and anonymous nature and confidentiality of the data analysis. They were also offered the opportunity to ask questions to the authors. In addition, permission was sought for the audio recording of the interview, so that it could be transcribed for further data analysis. In the case of underage participants, this informed consent was signed by their parents or guardians. Once the informed consents were signed and submitted, a day and an hour were agreed upon for the interview. The interviews were conducted by the first author between December of 2020 and February of 2021. They were recorded using a digital recorder and the audio files, as well as the transcriptions, were kept by the first author of the article. No one was able to access the content of the interviews except for the first author, and only the obtained information was shared with the research team. Given the pandemic crisis situation that arose as a result of COVID-19, the interviews were conducted in an online format using the Zoom platform. The interview was carried out by the first author without the presence of anyone else and it was recommended to be in a quiet place. Before the interview began, they were reminded of the purpose of the study and the ethical considerations such as that the results or information of the study that were potentially identifiable would be omitted (Jewett et al., 2019). In addition, participants were informed, both in the document of informed consent and verbally before the start of the interview, the right to request to stop recording at any time during the interview or to end it if requested (deleting the audio file). The interviews lasted an average of 52 min (range 40–65 min). All interviews were digitally recorded and *verbatim* transcribed.

### Data Analysis

A hierarchical content analysis of deductive-inductive nature was carried out. Following the directions of Sparkes and Smith (2014), the process followed a series of steps. In a first step of immersion, the authors read and re-read the interview transcripts, becoming familiar with the data. Categories were then identified and coded in the second step, those fragments of text that were interpreted as relevant issues in bullying experiences. In the third step, once the coding was done, the authors contrasted and connected the established categories by grouping them around three higher order themes that seemed to make sense to the participants, namely: (a) characterization of bullying, (b) dealing with bullying, and (c) consequences of bullying. The fourth step of this process consisted of re-examining the original transcripts, contrasting them with the structure of the themes and categories created from the authors’ interpretation, and discussing their coherence degree, as well as assessing whether they were missing to include any other relevant information. After reaching an agreement, the authors asked a critical friend, experienced in qualitative research, to review the analysis up to this point. This person agreed with the authors that the hierarchical content analysis entailed an adequate and accurate representation of the bullying experiences in youth sport training. The final phase of the process was the writing of the research report. Finally, it should be noted that this research followed the Reporting Standards for Qualitative Research (American Psychological Association [APA], 2020).

## Results

The results of this study are presented according to the three main themes established in the data analysis. First, a characterization of the bullying episodes experienced by the participants is described. Next, the used strategies and the most important social agents in dealing with bullying are presented. Finally, the short-term and long-term consequences of these experiences are shown. Table 2 summarizes the structure of the themes and categories around which the results are organized.

**TABLE 2:** Study themes and categories.

Bullying experiences in youth sport		
Characterization of bullying	Dealing with bullying	Consequences of bullying
- Bullying incidents	- Coping strategies	- Short-term consequences - Long-term consequences
- Bullying location	- The role of environmental agents	
- Justification of the received bullying		
- Simultaneity with school		

### Characterization of Bullying

The athletes suffered different types of intentional bullying aggressions. Roger highlighted the direct physical aggressions received during training, taking advantage of physical contact: “They did their best to try to foul me or hurt me […] when I ran with them, they pushed me or when I passed by the goal post [of the football pitch], they pushed me against the post.” Carla explained that these physical aggressions were constant by her teammates: “Whenever they could, they would give me a slap on the back of the neck.” Situations of indirect physical bullying were also mentioned: “My trainers disappeared once […] they put my backpack in the shower, sometimes they hid my match clothes […]. One day I caught a teammate cutting my T-shirt with scissors” (Anna). Victims also experienced acts of verbal bullying, such as receiving comments related to a lack of sport skills. According to Martí, these comments were intended to encourage him to leave the team and the competition: “They told me I was very bad, that I should leave the sport club, that they didn’t want me there, that I was useless.” Victor referred to the constant verbal aggression aimed at demoralizing him: “No matter what I did, they were like flies here [points at his ear]. They told me I was very bad, that I was shit.” Roger also explained that he received constant insults linked to physical characteristics: “They always made fun of my height.” In relation to social bullying, Carla explained how her teammates avoided sharing space and material with her: “They didn’t want to train in the same lane [of the pool] I was at, they didn’t share the material with me.” As for Noa, she commented on how the aggressors ignored her, both on and off the court: “They didn’t talk to me or they barely passed the ball to me when we played.” Diego referred to the spreading of false rumors as something that led to this ignorance: “They didn’t want to approach me because of what they had heard about me.” In Ivan’s case, he also explained the exclusion process he had experienced: “They started leaving me out and pushing me away […] they made plans without me and, in the end, they did everything without me until I was no longer part of the group.” In none of the participants’ accounts was referenced the experience of a cyberbullying situation.

The place where the assaults took place more frequently was the changing room. Anna recounted one of her experiences in this sport space, related with a physical assault on the victim: “One day we were in the shower, two teammates seized me and they cut a lock of my hair.” In this space, recurring actions with the intention of mocking and showing the victim up also happened, as explained by Martí:

Martí: They hid things from me in the changing room, and they did it often […] they also insulted me. They grabbed my T-shirt, walked out the changing room’s door, and started running. Then, I had to go out in my underwear to look for my things because I didn’t have my clothes.

Carla referred to the fact that the changing room is the place where her body was exposed, the target of the received attacks:

Carla: There [in the changing room], they started making fun of my body. Glances, whispers… They looked at me and commented in a quiet voice or said directly to my face: “What an arse, your breasts are growing…”. It was just when my body was changing.

In this way, this space becomes a place where the victim feels vulnerable because, as Gerard specified, the changing room was a space without surveillance or control by the coach and with no rules:

Gerard: The changing room is an enclosed space, where no one intervenes, where no one can see you, where there is no camera, where there are no rules. That can be the jungle. It is the one place where there is no coach figure.

David referred to the fact that when a changing room was not used, bullying did not occur: “It didn’t happen at parties because we didn’t take a shower afterward.” Bullying actions, however, were also carried out in spaces and moments beyond the changing room. This way, Anna, for example, commented on experiencing aggressions during the competition, when no one was looking: “On the competition bench, when everyone was watching the match, sometimes also something happened […] when no one was looking, that was the moment when they pulled a prank.” During training, bullying situations were also presented, for example, at the time to make groups, a critical moment for the victim, as Martí explained: “They made teams with a selection bias: I want you on my team, I don’t want you. And I was always left among the last ones.”

Participants justified the reasons why they felt they had been bullied in the sport environment. The victims’ references to their own physical appearance were recurrent, as Martí, who linked his obesity to the reason for being bullied, commented: “Everything happened because I was bigger, I was plump.” To the physical characteristics were added the psychological features, as Víctor explained: “Before, I was plump, tall. I was the easy target. And on top of that I was shy and didn’t say anything.” For his part, Diego perceived that one of the reasons why he received bullying was the visual impairment that he suffered:

Diego: I see very badly without glasses. Imagine swimming and having to communicate with others without seeing anything, all blurry […] this was object of ridicule, my teammates laughed at me when the coach told me off and I didn’t answer because I couldn’t see him. I turned around and, since I didn’t see the coach, that was object of ridicule.

In some cases, however, it was not about the physical appearance but the personality, like in the case of David: “I think they did it to me because of my personality, I’ve never been an easy person.” Sport skills were also one of the triggers for the received victimizations. It could be linked to possessing better sport skills compared to other athletes, which in the case of Noa, translated into playing more competition minutes than her teammates: “I think they did it to me because I played a lot, I played more than they did.” In the same way, it could be linked to a poor performance compared to the other members of the team, as in the case of Anna: “They were the stars and I was left behind.”

The bullying experienced at the sport club was sometimes experienced simultaneously with victimization at the school. Roger stated he had suffered this double victimization, referring to the proximity between the place where the extracurricular activity took place and the school:

Roger: The football pitch was 20 m away from school, you took ten steps and you arrived at the football pitch. It was right in front of the school. The players on my football team were almost all in my class, so the three of them that picked on me were both at school and at sport. I felt pressured at school and I also felt uneasy at football practice […] When I left school, I went to football practice and everything continued. At football practice the same thing happened to me again.

Like Roger, there are cases of other interviewees who also stated that the aggressors were the same in both contexts: “The aggressors were my high school classmates. They were not outsiders, but the same high school classmates who I also happened to meet at football practice” (Gerard); “In high school I also ran into them” (Carla); “The people who were bullying me in athletics also went to my school” (Laura). In Carla’s case, she explained how living this double victimization made the situation even more difficult to deal with:

Carla: We would go to school and they would bother me, we would go work out and they would bother me in training too. It was constant throughout the day and it wore me out. They were there all day long… I had these four classmates next to me who picked on me from six in the morning, when the first workout took place. Then, again at school… In the end, you end up feeling pretty bad. The constant teasing is very tough.

Laura explained the difference between bullying at school and in sport:

Laura: At school this [bullying] happened for long periods of time. They had plenty of time to do whatever they wanted to me. On the other hand, in athletics they only had the time that the training lasted. […] It was little time compared to the time they had at school.

Roger commented that this difference between the two environments even made him think that it might not be bullying what he experienced in the sport context: “At school that was very insistent, it was daily, it was every day. At football practice, as it was 2 days a week, I couldn’t quite relate it to bullying.” Diego, who had also experienced this double victimization, mentioned the differences in terms of the referents in both contexts, being the figure of the teacher an agent in whom most trust was placed rather than with the coach: “With the school teacher there is a greater level of trust, it’s easier to tell something to the teacher than to the coach.” During his interview, despite stating that he did not suffer from bullying at school, Ivan presented his personal perception toward the difference between the contexts, based on the obligatory nature or the willingness that characterizes both environments:

Ivan: I think it’s worse in school. You can’t leave school. At school you spend 6 h doing things you might not like, with people you don’t like and… of course, imagine… In sport you can always change sport clubs, you can try another sport, you can train by yourself individually, you can do whatever you want with sport. But you can’t quit school.

### Dealing With Bullying

Regarding the different coping strategies used by the victims to respond to the problem, the law of silence appeared, as in the case of Laura, who stated that she did not want to explain it to anyone: “I was silent, I preferred not to say it, I kept it for myself […] it affected me, but I tried to hold it in.” There were several reasons why the victims kept silent, but the most predominant one was the fear of the consequences that saying something could lead to: “Because they would make things even worse for me” (Roger); “I was afraid they would go even further” (David); “I was afraid of what they might think of or what they might do” (Ivan); “For fear of what might happen next” (Martí). On the other hand, the victims explained they lived the situation with resignation. Thus, Diego commented: “In the end it is also a matter of getting used to it. If you’re used to being told the same thing more or less every week, it’s the same as being told again.” One of the strategies was also to avoid the aggressors, as Gerard explained: “When I saw they [the aggressors] wanted to harm me and bother me, I avoided contact, I tried not to look at them.” However, Gerard commented that, at the same time, in response to one of the received aggressions, he reacted by confronting the aggressor, because the situation overwhelmed him: “One day they wouldn’t stop bothering me in the shower, I grabbed them and told them: I can’t stand this anymore, stop.” Still, he commented on the ineffectiveness of his response. On the other hand, some victims expressed the difficulty of getting out of the situation in which they were caught up in, like Martí, who made explicit reference to the process of helplessness experienced: “There was no way out of there. It was a loop.”

When the victims explained their experiences, they referred to the importance of the role played by some agents in the sport environment. One of these agents where the bystanders (teammates), who did not always react by helping the victim out when they witnessed the bullying situation. In Martí’s case, he explained that he had the perception that the bystanders were afraid of being harmed in the event that they helped him: “They looked and avoided getting into trouble. They saw I wasn’t feeling good there, but in the end, my teammates avoided getting into trouble.” They could also side with the aggressor, supporting him by laughing, as David stated: “If I was taking a shower and they soaked my trainers, the rest would laugh. They sided with the aggressor. They laughed at their pranks.” Even Gerard commented: “The rest of the teammates took it as a joke.” In Laura’s case, the bystanders acted in favor of the victim, communicating the situation to their respective families: “Thanks to them [the bystanders] my mum found out that this was happening.” In relation to the latter aspect, families also play a very important role. Despite it, some commented that they did not dare to comment on it to the family, like in the case of Martí: “I didn’t want to tell anyone… and when I say anyone, I mean anyone. Not even my parents.” Even Ivan commented that up to the present date, he had never spoken with his family about this subject: “To this day I have never told them anything either”; and Roger confessed that he currently believed that his family did not know the magnitude of the problem: “My family knew that I didn’t get along with this boy [the aggressor], that I had removed myself from the group, but they didn’t imagine that I suffered that much.” Nonetheless, there were cases in which the victims did tell to the families, who tried to provide solutions in this regard. In the case of David and Anna, they were suggested to abandon that sport: “I talked to my parents and they told me to leave” (David); “My family told me to leave” (Anna). In Carla’s case, once the family found out about the problem, they suggested she changed sport clubs: “From that moment onward, my mum supported me more and told me we had to do something about it. We looked at other sport clubs where I could go.” However, there were families that, when they found out about the problem, decided to solve it by themselves with the other families, as in the case of Victor and Anna: “Until I mentioned it at home… And then yes, my parents told their parents and the issue was solved” (Víctor); “The parents acted, they talked to each other” (Anna). In Laura’s case, she explained that when the family figured it out, they went straight to the sport organization to deal with the problem: “When they found out, they addressed it from the inside [within the sport organization].” The victims’ silence could sometimes be justified by a lack of communication with another of the important agents for dealing with the phenomenon: the coach. Roger explained: “I was pretty cold with the coach; I didn’t talk too much to him. And I had a curt manner. We never ended up talking about anything… We just talked about football related stuff and that’s about it.” The feeling of the athletes was that the coach acted only with the aim of winning matches and that they did not dedicate enough time to the relationship or personal aspects of their players, as Martí stated: “His conduct was always more technical, to make the game run smoothly and not waste time on these issues.” In relation to the coach’s actions in the face of the bullying situation, a lack of intervention on their behalf was observed. Martí, for example, detected passivity in the coach, who showed little predisposition to change the existing mindset: “I never saw a reaction willing to break down this bad relationship that was there.” Anna expressed how the coach trivialized the problem and did not give importance to what she explained:

Anna: “He always said the same: you’re young, you’re young, you’re playing… these are youngsters’ things […] I told my coach that they didn’t want to pass me the ball and he answered that all I had to do was work harder.”

Ivan, in his case, explained that when the coach witnessed situations of violence, he addressed the aggressors to no avail: “At most he told them [the aggressors]: “don’t do this”, but they kept doing it. It was useless.”

### Consequences of Bullying

Experiencing a bullying situation directly affected the daily lives of the victims. There were different feelings and emotions generated as a result of the situation experienced at the time of the victimization. In general, suffering was reflected, as Martí and Roger showed: “It was hell, it was the worst feeling I could experience” (Martí); “It was horrible” (Roger). Sadness was a recurring feeling. Carla explained: “I got home very sad”; and Anna: “I got home and I cried…”. Anger and helplessness also developed in the interviewees, as Roger called it, due to the imbalance of power he perceived: “At the time I felt a lot of anger, also helplessness, because they were the tall ones, they were a group.” Due to the exclusion and rejection by his teammates, Víctor commented that he had the feeling he was being a nuisance to the group: “I had the feeling that I was annoying. Of course, always being rejected leads to the feeling that you’re bothering everyone.” Related to the latter aspect, Martí also commented that he had the feeling of not being loved, linked to a feeling of loneliness: “At that sport club, I felt awful. I was like one of those old balls you keep in the corner.” Anxiety was also something present on a daily basis during bullying episodes. Martí, for example, related this distressing feeling to the ignorance of what could await him on training days: “I went training and I thought: let’s see what awaits me today.” In Noa’s case, this anguish affected her health directly, leading to anxiety attacks:

Noa: Yes, it was anxiety, it was anxiety. I couldn’t breathe… It was the worst, because I was there and I felt like I couldn’t breathe. I stopped for a moment, tried to catch my breath and I couldn’t get it into my lungs… It was horrible, it was a horrible situation […] I couldn’t even last 5 min on the court.

According to Ivan, experiencing a bullying episode affected his motivation and concentration, elements that directly conditioned his performance in sport: “I did everything reluctantly […] I was very distracted, my concentration level was very low, I didn’t know any plays… My performance dropped a lot.” Athletic performance could also be affected by low self-confidence and embarrassment when training or competing, like in the case of David and Víctor: “I always felt very insecure about carrying out any action and I was embarrassed as well. I think it all comes down to wanting to do everything right in order to not screw up” (David); “In the end you lack confidence when you play, you think you’re shit at what you do” (Víctor). Anna even though she wasn’t good enough to play that sport: “Maybe I’m not good at it, maybe I’m not as good as them.” Ivan and Roger referred to the fact that they stopped enjoying the sport they practiced due to the received bullying and, in Roger’s case, he specified he adopted a constant hypervigilant attitude: “I just stopped enjoying basketball, which was the sport I’d played my whole life” (Ivan); “I didn’t enjoy the matches… I was thinking about what they’d do to me, thinking about various things… I wasn’t happy there” (Roger). Not enjoying the sport sometimes translated into a lack of motivation, as Carla commented: “I didn’t want to go to training, I didn’t feel like it.” In Martí’s case, the bullying he received led him to abandon the sport he practiced (football). He commented on his desire not to be part of any other team and to change from a team sport to an individual sport, where he didn’t need to interact with other teammates:

Martí: I was on the verge of not wanting to play football anymore because of that […]. I decided to leave the sport club and from there I looked for individual sports. I liked tennis and I wanted to practice an individual sport, where everything depended on me and I didn’t have to socialize with anyone. I decided to distance myself from the “team” issue for a while […] I gave up football and I had no intention of ever going back. I wanted to see football as a spectator.

Although Martí was the only interviewee who had abandoned the sport, the victims commented that they had considered giving it up as well, as Ivan explained: “I thought about quitting plenty of times, I wasn’t feeling good there.” In Carla’s case, she commented on how the bullying she received also discouraged her from going to school: “I thought about dropping out of school, about leaving everything behind.” In Noa’s case, she referred to the fact that the consequences were not only reduced to a personal level, but also group consequences emerged, affecting the whole collective:

Noa: “In the end it broke me, but it also broke up the team. There was so much shit accumulated in that circle, that in the end it was destroyed. It was like a time bomb, and that bomb finally exploded. In the end, everyone left for different sport clubs.”

The bullying situations experienced not only affected the victims at the time in which they lived through them, but also in the long-term. In this sense, Gerard commented: “I think this has lifelong consequences”; and Carla: “It leaves a mark for life […] it’s like having a chip on one’s shoulder. I think it will be there forever.” Carla also expressed her fear of living the same thing again: “You’re afraid that people will really get to know you and that they will hurt you, because they’ve done it to you before.” Thus, some consequences are very present in the lives of the interviewees. In that sense, Noa, reliving the situations she experienced, explained that she still had a hard time describing what she went through, to the point of having to stop the interview on different occasions: “I can talk about it, but it’s hard for me [cries].” Some interviewees referred to the fact that, as of today, due to the situation they had experienced, they consider that they have certain difficulties linked to having appropriate social skills. In this way, Roger commented that it was difficult for him to interact with any large group of people: “I find it very hard to open up to large groups. First, I have to know the group well and then I can open up… or not.” Ivan explained: “As a result, I started developing more confidence problems, it was harder for me to interact with people.” Laura also commented that she was currently somewhat afraid of what people might think of her: “I’m scared of interacting with new people: what will they think of me? What will they say to me?” And Gerard explained that because of what he went through, he was always alert, trying to be accepted by the group: “I’m always trying to get people to like me.” Carla and Roger referred to the changes in their personality: “Before I was a very outgoing person, I interacted with everyone… Now I interact with everyone but I consider myself to be shy, very shy” (Carla); “I didn’t use to be as shy as I am now” (Roger). Problems related to self-esteem were also expressed, which persisted over time: “It has affected me in many ways, especially my self-esteem. With issues like when it comes to flirting, when it comes to everything… Because I think I have a much lower self-esteem” (Carla). Although most of the interviewees spoke of long-term negative consequences, reference was also made to aspects such as the empathy they have developed as a result of being victims and which makes them aware of the importance of acting in situations of bullying, as Martí stated: “It has helped me learn. I learnt what I wouldn’t like to be done to me, and not to do it to anyone. If I see someone doing it, I try to help” (Martí).

## Discussion

Although the presence of bullying in youth sport is well known, being a topical issue necessary to address (Mishna et al., 2019; Nery et al., 2019; Vveinhardt et al., 2019a), there is a lack of literature that has taken on the analysis of the experiences of those athletes who were victims in their childhood and/or adolescence. Thus, this study aimed to know the experiences of bullying in athletes who had been victims during their youth sport training. The results are structured in three main themes: characterization of bullying, dealing with bullying and consequences of bullying. Bullying situations involved behaviors included in the typologies of physical, social and verbal bullying. The physical forms of direct aggression (for example, hitting) were present among some of the interviewees and, while these actions could be masked in the very dynamics involved in the sport practice (the game itself), other indirect behaviors appeared (such as stealing or damaging personal items). Victims commented, as observed by Kerr et al. (2016), being excluded and ignored by the group (social bullying). Verbal aggressions were present and, above all, referred to the victims’ lack of sport skills, in line with the study by Kentel and McHugh (2015) with aboriginal women athletes. No cyberbullying experience emerged, following the line of other studies which showed that this typology was the least common in the sport environment (Nery et al., 2019). The quintessential space where bullying episodes took place was the changing room (Fisher and Dzikus, 2010; Nery et al., 2019). In the same way that Herrick and Duncan (2020) observed, the changing room becomes a space for comments related to the body, and some of the victims stated that when there was no opportunity to share this space with their teammates, the aggressions did not occur. Bascón-Seda and Ramírez-Macías (2020) observed that victims of bullying in physical education did not enter the toilets and changing rooms precisely to avoid these situations. The athletes themselves defined this space as a place where they spent time with their equals, where there was no presence nor supervision by the coach, which explains the perception of danger due to the development of bullying episodes in this space (Volk and Lagzdins, 2009; Kerr et al., 2016; Nery et al., 2019). For this reason, it is necessary to develop a control plan for the situations that may arise in the changing room, either directly by adults, or indirectly, by the collaboration and participation of any member of the team that the coach trusts, such as the captain (Nery et al., 2020).

In relation to the reason why the victims considered they were bullied, in line with Forsberg and Horton (2020), attributions of an internal nature, of self-blame and linked to issues related to skills, character, physical characteristics and with presenting some type of disability were observed. This internal attribution of the situation (self-blame) may lead to a higher probability of continuing to receive bullying and the appearance of depressive symptoms, making an intervention linked to attributional change necessary (Schacter et al., 2015). In relation to the perception of possessing poor sport skills, our results concur with those of Mishna et al. (2019) and with Kerr et al. (2016) regarding that, when a member of the group did not have great sport skills, it could negatively affect the overall performance of the team and, thus, increase the chances of being bullied. Differences in motor skills lead to an imbalance of power (Nery et al., 2020) and giving too much importance to the result in team sports can become a catalyst in the emergence of bullying behavior. Despite a lack of sport skills is a relevant trigger when it comes to suffering from bullying, some of the victims claimed to have been bullied for showing superior sport skills compared to the rest of their teammates. Following the Social Comparison Theory by Festinger (1954) and, in the same way it occurs in the educational environment, especially among boys (Bergold et al., 2020), some athletes who become aggressors may feel threatened and jealous of those with better sport skills (Forsberg and Horton, 2020). Victims also explained that personality traits such as shyness were also a reason to become a victim, since this leads them to have less ability to defend their own physical and psychological integrity (Hernández and Saravia, 2016). On the other hand and in line with the results of Gardella et al. (2020), physical appearance, such as height, obesity and not having an athletic body were also some of the elements that stood out for receiving bullying actions. Hill (2015) suggested that boys who were more athletic and had a more positive view of their bodies were more likely to be popular, while those who did not fit below the standard were rejected, making them more vulnerable to being bullied (Kerr et al., 2016). Suffering from certain types of disability also increases the chances of being bullied at school (Pinquart, 2017) and in the specific context of physical education (Ball et al., 2021), as well as presenting an attribution of the bullying situation linked to the disability or being perceived by themselves as a different player (Danes-Staples et al., 2013).

On the other hand, some of the participants presented simultaneous victimization at the sport club and at school, in line with other research indicating a continuity in victimization (Collot and Dudink, 2010; Vveinhardt and Fominiene, 2019). The extracurricular sport organization was often linked to the school itself or the sport practice was carried out in private sport clubs near the center. The victims also referred to the difference in time and intensity spent at school and at extracurricular sport activities. This meant that at school not only was there a greater probability of interaction with the aggressors, but also that the victims could underestimate and/or have difficulty recognizing the bullying situation they were experiencing in the sport environment. At the same time they commented, in line with the work of Nery et al. (2020), that schooling is compulsory and practicing sport is voluntary, allowing them to not continue and abandon it. In this sense, it is believed important to have an ecological perspective of the phenomenon of bullying (Espelage, 2014) and to promote dynamics to favor communication between school and non-school environments and entities where the child or adolescent can participate.

Regarding the different coping strategies used by the victims, it was observed that in general, and in line with the results of Nery et al. (2020), the most common was the use of passive strategies, linked to emotions (fear, resignation, etc.), rather than active strategies (asking for help or confronting the aggressor). The fact of not asking for help from their environment (family, teammates, and/or sport staff) can be explained because historically the sport setting identifies the action of asking for help as a sign of weakness on the athlete’s part (Stirling et al., 2011) and of shame (Mierzwinski and Velija, 2020), encouraging the athlete to mask the situation (Newman et al., 2021). The perception of fear of retaliation by the aggressor was also present among the victims and as an explanation for the passive action of the teammates (Flores et al., 2020; Nery et al., 2020). This facilitates the continuity of bullying over time (Collot and Dudink, 2010). On the other hand, resignation or getting used to the experienced situation were also present among the victims of the study. This passive response may be a condition, as observed by Steinfeldt et al. (2012), for creating and maintaining an environment with little moral culture within the group and a high compliance with traditionally masculine rules in sport. Another used strategy was to avoid the aggressor and, in some cases, to confront him directly as a way of self-protection, being perhaps the only and last resort that victims have to end the situation (Evans et al., 2017). As in the retrospective study with victims of bullying in the educational context by Tolmatcheff et al. (2019), the strategies related to maintaining a direct confrontation with the aggressor or presenting an avoidance attitude did not give positive results to eliminate bullying.

Understanding bullying as a social phenomenon (Salmivalli et al., 1996) requires consideration of the role that different agents in the sport environment play in bullying situations (Shannon, 2013). The victims expressed a perception of the importance of their equals’ action in the maintenance and development of bullying episodes, showing a sense of lack of support. This lack of support could be related to the fact that their equals do not know what to do about the situation, having the perception that if an adult is informed, the situation will not be resolved (Bauman et al., 2020) or fearing that they might also become victims (O’Connor and Graber, 2014; Flores et al., 2020; Jeckell et al., 2020). One of the victims reported the importance of their equals not only preventing the emergence of bullying behaviors, but also their intervention, stopping the situation (Tolmatcheff et al., 2019; Nery et al., 2020). However, it must be borne in mind that when trying to defend the victim, the defenders themselves may suffer negative effects on their mental health, such as anxiety or depression (Wu et al., 2016). In this sense, it is important to create dynamics to reinforce the development of empathetic and prosocial behaviors among young bystanders, to have clear indications on what to do, and to create environments that allow complaints in the presence of bullying situations (Nery et al., 2020). Regarding the role of the family, it should be highlighted that, in line with what Nery et al. (2019) had already found out, the victims rarely asked the families for help. In fact, some of the interviewees stated that, to date, they had not verbalized the situation of bullying they had experienced to their families. It is difficult for the families to know that a bullying situation occurs if their child hides it from them and, in many cases, the victims hide it out of shame, due to the perception that the problem is not so serious or the feeling that informing their family would make the bullying situation even worse (Larrañaga et al., 2018). Some interviewees broke their silence by informing their families of the situation and the strategies they adopted were varied: making their child change sport clubs, talking directly with the families of the aggressor or aggressors or with the sport organization. Families do not have clear indications of how to act when a case of bullying occurs, reason why it would be necessary to promote their training (Flores et al., 2020). On the other hand, the coach becomes one of the key agents, since they can act as a role model or mentor and promote, maintain or inhibit bullying (Nery et al., 2020; Vveinhardt and Fominiene, 2020). The victims perceived that the coach had little interest in personal experiences, giving little importance to the interpersonal relationships created in the athletes’ group and, at the same time, trivializing the situations of conflict that could arise, focusing their intervention in merely sporting elements. In line with our results and in the school context Di Stasio et al. (2016) observed that in environments where competition and social comparison between students are fostered, as well as in those environments with a poor teacher-student relationship, bullying situations are more common. A lack of teacher-student relationship can lead victims to stop reporting the event due to a perception of indifference from the teacher, or due to the feeling that they will not solve the problem positively (Nery et al., 2020). Therefore, the actions taken by the coaches did not favor, in general, putting an end to bullying, probably due to a lack of education and knowledge about this phenomenon (Kowalski, 2017; Flores et al., 2020) and due to their education being focused on athletic performance, with traditional training styles (Vveinhardt and Fominiene, 2020), creating a high pressure environment for the victim (Vveinhardt et al., 2017). Thus, it is advisable to increase the knowledge on the subject by the coaches and articulate pedagogic dynamics that are in line with the athletes’ youth sport training (Collot and Dudink, 2010; Baar and Wubbels, 2013; Shannon, 2013; Nery et al., 2019; Flores et al., 2020).

Regarding the consequences of suffering bullying, negative emotional effects were observed among athletes (Kentel and McHugh, 2015; Jewett et al., 2019; Vveinhardt et al., 2019a). Sadness was a recurring feeling in the daily lives of the victims (Monks et al., 2009; Hutson, 2018) as was the feeling of helplessness at not being able to find a solution to the problem (Side and Johnson, 2014). Often, there was an attitude of hypervigilance (Evans et al., 2017), distress and the onset of anxiety attacks, probably as a response to the stress caused by the humiliation to which they are subjected (Hernández and Saravia, 2016). It also directly affected the perceived athletic performance, linking it to a loss of confidence (Vveinhardt et al., 2019a), shame, and decreased self-esteem (Jewett et al., 2019), ceasing to enjoy the sport that had previously brought them positive experiences (Kentel and McHugh, 2015; Evans et al., 2017; Jewett et al., 2019). Abandonment of the sport seeking to get away from people who bullied them also becomes a very likely consequence (Kentel and McHugh, 2015; Stefaniuk and Bridel, 2018; Mishna et al., 2019; Nery et al., 2020). In our study, victims did not make explicit reference to a decrease in their academic performance, although in previous studies it has been observed that victimization can have an impact on the ability to focus on academic demands (Jewett et al., 2019; Vveinhardt et al., 2019a). At the same time, it must be borne in mind that consequences are not reduced to the victim, but the consequences are also generated at group level, creating an environment of little cooperation and cohesion (Vveinhardt et al., 2017; Jewett et al., 2019). As it happens in the school environment, becoming a victim of bullying in sport during their youth sport training implies negative consequences that go beyond the period of time in which the victimization actually takes place (Wolke and Lereya, 2015; Camodeca and Nava, 2020). The interviewees commented on the fear of reliving those situations, the difficulties of establishing social relationships and the changes that had taken place in their personalities. As opposed to the study by Vveinhardt et al. (2019a), the victims did not show “positive” consequences of the situation in the long term (being bullied does not make the victim “stronger”), but stated that the experience had fostered a strong sense of empathy toward situations of bullying that may occur in their environment. Moreover, although victimization in the school setting may facilitate substance abuse behaviors (Moore et al., 2017) in our study it was not mentioned by any of the participants, probably due to the fact that people who spend more time practicing sport have a lower frequency of consumption (Schmidt et al., 2019).

## Conclusion

The aim of this study was to find out, in retrospect, the experiences of athletes who had been bullied in the sport context during their youth sport training. The analysis makes it possible to identify the presence of physical, verbal and social bullying in the sport environment, the changing room as the place where bullying situations are most frequent and an internal attribution (self-blame) of the phenomenon related to motor skills and the physical and psychological characteristics of the victim. In addition, it has been possible to observe the presence of a double victimization, at the sport club and at the educational center, the use of passive strategies to deal with the situation, the little support perceived by the victims from the sport agents (teammates and coach) and the negative effects of having been bullied, in the short and long term and at a psychosocial level. Given the results, and from an ecological perspective of the problem (Espelage, 2014), it becomes important to raise awareness and train the entire sport community -teammates, coaches, management teams, and families- on the subject of bullying (Nery et al., 2020). It is necessary to design actions in sport organizations to prevent it, recognize possible signs of bullying situations and develop action protocols in the face of bullying to promote the creation of safe, non-violent and respectful sport environments based on respect and equity (Mountjoy et al., 2016; Vveinhardt et al., 2019a). It is necessary to indicate some limitations of the study and guidelines for future research lines. Although the sample includes victims of bullying from different sport categories, there is a greater presence of team sports than individual sports, specifically from basketball and football. We believe it necessary to carry out specific studies of a certain sport category or of the same group of sports in the future, as well as comparative analyses in relation to gender, sport groups and/or countries. At the same time, it is considered interesting to analyze the health consequences of being a victim of bullying in sport and the effect it has had on the practice of physical sport activities in the longer term (middle age).

## Data Availability Statement

The raw data supporting the conclusions of this article will be made available by the authors, without undue reservation.

## Ethics Statement

The studies involving human participants were reviewed and approved by Ethics Committee of the Catalan Government’s Sports Council (009/CEICGC/2021). Written informed consent to participate in this study was provided by the participants’ legal guardian/next of kin.

## Author Contributions

XR, CV, and PM: study design, data analysis, and manuscript preparation. XR: data collection. CV: conceptualization and funding procurement. All authors have read and agreed to the published version of the manuscript.

## Conflict of Interest

The authors declare that the research was conducted in the absence of any commercial or financial relationships that could be construed as a potential conflict of interest.

## Publisher’s Note

All claims expressed in this article are solely those of the authors and do not necessarily represent those of their affiliated organizations, or those of the publisher, the editors and the reviewers. Any product that may be evaluated in this article, or claim that may be made by its manufacturer, is not guaranteed or endorsed by the publisher.
